# Combined Use of Cell Block and Smear Improves the Cytological Diagnosis of Malignancy in Non-Palpable Breast Lesions Screened by Imaging

**DOI:** 10.1155/2023/1869858

**Published:** 2023-05-03

**Authors:** Rieko Nishimura, Mikinao Oiwa

**Affiliations:** ^1^Department of Pathology, Nagoya Medical Center, Nagoya, Aichi, Japan; ^2^Department of Radiology, Nagoya Medical Center, Nagoya, Aichi, Japan

## Abstract

**Background:**

Currently, core needle biopsy is replacing fine needle aspiration biopsy (FNAB) for pathological diagnosis of breast lesions. However, FNAB is extensively used for diagnosing breast lesions, including screened lesions, at our hospital. Furthermore, direct smears as well as cell blocks (CBs) from the FNAB specimens have been used. To prepare the CBs, hematoxylin and eosin (HE) staining as well as immunostaining with a mixture of p63 and cytokeratin 5/6 antibodies are routinely used. Therefore, in the current study, we sought to assess the efficacy of diagnosing breast lesions using conventional smears and CB immunostaining.

**Methods:**

Breast FNAB reports of direct smears and CBs from The Nagoya Medical Center between December 2014 and March 2020, were reviewed. The efficiency of diagnoses made with direct smears and CBs were compared using histology-based diagnoses.

**Results:**

Among the 169 histologically confirmed malignant lesions, 12 lesions that were reported as unsatisfactory, benign, or atypia probably benign, using direct smears were diagnosed as malignant using CB. Histologically, these lesions were diagnosed as carcinomas with mild atypia or papillary structures. Ten (83.3%) of the twelve lesions were non-palpable and only detected upon imaging.

**Conclusion:**

Combined use of CB and conventional smear leads to the detection of more malignant lesions in breast FNAB specimens, particularly in lesions detected by imaging alone. Immunostaining of CB sections using a mixture of p63 and cytokeratin 5/6 antibodies provides more information than HE staining alone. Breast FNAB with CB preparation can be successfully applied for evaluation of breast lesions in developed countries.

## 1. Introduction

For several decades, fine needle aspiration biopsy (FNAB) has been used as the method of choice for pathological diagnosis of breast lesions. However, over the past decade, core needle biopsy (CNB) has gradually replaced FNAB, particularly within developed countries, including Japan [1]. Compared to CNB, the use of FNAB for breast lesions has several limitations with respect to detection of cancer invasion, classification of proliferative lesions, including ductal hyperplasia and papilloma, and diagnosis based on poor quality smears [2]. However, preparation of cell blocks (CBs) using cells remaining after smear preparation appears to have overcome these limitations [3]. This might be due to CB slides retaining the histological architecture, making them appropriate for immunostaining. In addition, CBs can be stored in the same way as histology blocks and can be used for preparing additional sections for further examinations. Indeed, a Nigerian prospective study reported that the use of CBs, together with smears, markedly improved the diagnostic potential of breast FNAB for palpable masses by minimizing the risk of atypical and suspicious diagnoses for malignancy [4].

The Nagoya Medical Center is a general hospital with 740 beds situated within a large city in Japan. To date, the hospital has relied heavily on FNAB for the evaluation of breast lesions, including lesions screened by imaging, with CBs routinely prepared from the breast FNAB specimens in addition to conventional smears since December 2014. Moreover, pathologists and technicians at this hospital confirmed that double immunostaining with p63 and cytokeratin 5/6 antibodies effectively distinguishes borderline papillary lesions of the breast on histological sections [5]. Hence, breast CNB sections are now routinely immunostained within this medical center. Moreover, along with hematoxylin and eosin (HE) staining, double immunostaining with p63 and cytokeratin 5/6 antibodies is routinely performed for all CB sections to detect myoepithelial cells and monoclonal proliferation of epithelial cells. However, reports on the effectiveness of combining conventional direct smears and CB preparation, with immunostaining of breast FNAB specimens, including screened lesions, at a general hospital in a developed country, are lacking.

Therefore, in this study, we assess the techniques used for the management of breast FNAB specimens at our hospital and report the advantages of combining CB and conventional smears as a routine procedure. Furthermore, we discuss the importance of using FNAB in pathological diagnosis of breast lesions in developed countries.

## 2. Materials and Methods

### 2.1. Lesions and Specimens

Breast FNAB reports made at the Nagoya Medical Center, between December 2014 and March 2020, were reviewed. Lesions with both conventional direct smears and CBs were selected for analysis. The lesions included not only palpable masses but also non-palpable lesions detected in screening. Among these lesions, those that were diagnosed histologically were included in the study. Among the lesions confirmed as malignant lesions based on histology, those that were not found to be malignant in direct smears but were diagnosed to be malignant using CB were characterized.

### 2.2. Data Collection

The data on the lesions included in this retrospective study were collected from the hospital's electronic pathology reporting system. This included the data from the cytology smear report (category, cytological findings, and cytological diagnosis), CB report (diagnosis, immunohistochemistry [IHC] findings), and histological report (diagnosis, IHC findings).

### 2.3. Fine Needle Aspiration Biopsy

Usually, a single specimen was aspirated from each lesion using a 20- or 22-gauge needle with an extension tube attached to a 20 mL syringe. The aspirated specimen was flushed into 10% buffered formalin in a specimen bottle to prepare CBs. Then, the extension tube was removed, and the residual specimen at the neck of the syringe was smeared on a glass slide to obtain a direct smear. A second aspiration was performed if the amount of aspirated specimen was insufficient for generating both CB and smear. In which case, the first aspiration was used for CB, and the second aspiration was used for the direct smear in the current setting.

### 2.4. Preparation of CB

CBs were prepared according to the protocol described in our previous studies [6]. Briefly, the specimens in 10% buffered formalin were fixed for 6–48 hours, processed for CB preparation using the sodium alginate method, and embedded in paraffin. In brief, the sample-containing tubes were centrifuged at 3,000 rpm for 5 minutes to remove formalin, following which 1% sodium alginate was added, the tubes were recentrifuged at 3,000 rpm for 5 minutes, and 1 M calcium chloride was added. The gel pellet formed during this process was embedded in paraffin, and the paraffin CBs were then processed in the same way as histological specimens.

### 2.5. Staining of the CB Sections

Sections prepared from all CBs were routinely stained with HE and IHC antibodies. The Ventana BenchMark system (Roche Diagnostics, Basel, Switzerland) was used for IHC. A mixture of equal amounts of p63 and cytokeratin 5/6 (Roche Diagnostics) antibodies was used to detect myoepithelial cells and monoclonal proliferation of epithelial cells [5].

### 2.6. Preparation and Reporting of Cytology Smears

The direct smears were alcohol-fixed and treated with Papanicolaou stain. The results obtained using the smears were reported using a five-category system based on the guidelines for non-operative diagnostic procedures and reporting in breast cancer screening published by the National Health Service Cancer Screening Programmes, proposed in the UK in 2001 [7]. The five categories were as follows: unsatisfactory, benign, atypia probably benign, suspicious of malignancy, and malignant. The criteria used for defining the categories were similar to those used by the International Academy of Cytology Yokohama System for Reporting Breast Fine Needle Aspiration Biopsy Cytopathology, proposed in 2019 [8].

### 2.7. Categorization of CB Results

CB results were originally reported with diagnosis and descriptions. These were categorized into unsatisfactory, benign, indeterminate, and malignant.

### 2.8. Categorization of Histology Results

Histology results were originally reported with diagnosis and descriptions. These were categorized into benign, borderline, and malignant. Borderline histology included borderline phyllodes, tumor, and atypical ductal hyperplasia.

## 3. Results

During the study period, 251 CBs were prepared from 1,806 breast FNAB specimens. Among these 251 specimens, smears were prepared from 247, among which, the corresponding histological diagnoses of the lesions were available for 187 specimens. Ultimately, 169 were confirmed to be malignant, 14 as benign, and 4 as borderline based on lesional histology. Of the 169 histologically malignant specimens, 129 (76.3%) were diagnosed as suspicious of malignancy or malignant using direct smear (1), and 103 (60.9%) were diagnosed as malignant using CB (Table 2). The number of unsatisfactory reports for lesions found to be histologically malignant was significantly higher when CB was used (38 specimens, 22.5%; Table 2) than when direct smears were used (10 specimens, 5.9%; Table 1).

**Figure 1:**
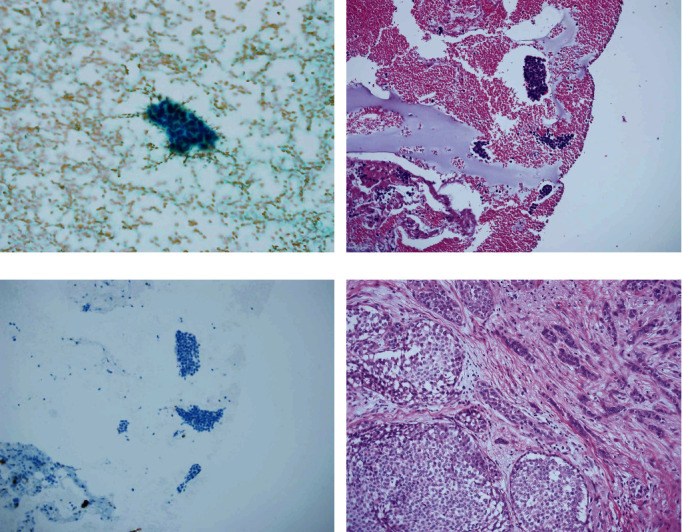
A leasion with unsatisfactory smear confirmed to be malignant using a cell block. (a) Conventional smear showing few small clusters of epithelial cells in a bloody background. The epithelial cells had small- to moderate-sized nuclei with conspicuous nucleoli (Papanicolaou stain). The smear was reported to be unsatisfactory due to the low number of epithelial cells. (b) Cell block slide showing several small clusters of epithelial cells in a bloody background. The clusters were packed with monotonous cells with mild atypia (hematoxylin and eosin [HE] stain). (c) Serial section of epithelial cells was negative for p63 and cytokeratin 5/6 (immunohistochemistry). The specimen was confirmed to be malignant. (d) Histology of the resected lesion. The lesion was histologically diagnosed as invasive lobular carcinoma (HE stain).

**Figure 2:**
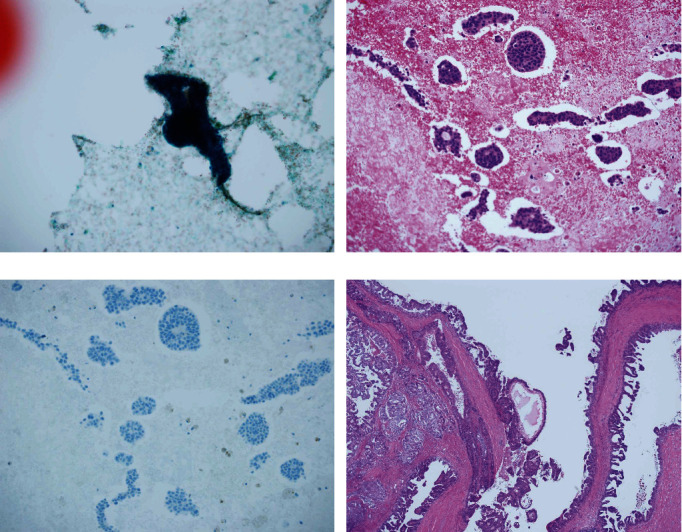
An “atypia probably benign” lesion smear confirmed to be malignant using a cell block. (a) Conventional smear showing few clusters of epithelial cells in a bloody background with histiocytes. Myoepithelial cells were not found in the clusters. The epithelial cells had small nuclei with high chromatin staining (Papanicolaou stain). Cystic lesion with ductal cell hyperplasia was suspected. The smear was reported to belong to the “atypia probably benign” category. (b) Cell block slide showing several epithelial cell clusters with papillary or tubular structures in a bloody background. The epithelial cells showed moderate nuclear atypia (HE stain). (c) Serial section of epithelial cells was negative for cytokeratin 5/6, and myoepithelial cells beneath the epithelial cells (p63 and cytokeratin 5/6, immunohistochemistry) were absent. Carcinoma with cyst formation was suspected. (d) Histology of the resected lesion. Atypical cells lining the inside of cyst wall and focal invasion were observed at the wall. The lesion was histologically diagnosed as encapsulated papillary carcinoma with invasion (HE stain).

Out of the 169 histologically confirmed malignant lesions, 12 that were reported to be unsatisfactory, benign, or atypia probably benign, using conventional direct smears, were diagnosed as malignant using CB (Table 3). The histological diagnoses of these 12 lesions were as follows: one each of invasive lobular carcinoma (Figure 1), invasive breast carcinoma of no special type (IBC with NST), ductal carcinoma in situ (DCIS), mucinous carcinoma in the four unsatisfactory smears, IBC with NST in one benign smear, two IBCs with NST, two DCIS, one encapsulated carcinoma, one encapsulated papillary carcinoma with invasion (Figure 2), and one papilloma with DCIS in the seven atypia probably benign smears.

The clinical findings associated with these 169 lesions were as follows: 85 (50.3%) palpable masses, 79 (46.7%) detected using imaging, and 5 (3.0%) had nipple discharge. Among the 79 lesions detected using imaging, 51 (64.6%) were detected using breast screening. Ten (83.3%) out of the twelve lesions, not reported as malignant using conventional smear but confirmed to be malignant using CB, were only detected using imaging (Table 4). Eight out of the ten lesions were detected by breast screening, one was detected during systemic check-up after surgically removing the malignant tumor, and one was detected using imaging for contralateral breast pain.

## 4. Discussion

The Nagoya Medical Center is a general hospital in Japan where the use of breast FNAB is gradually decreasing. Here, by reviewing breast FNAB data over 5 years and 4 months, we elucidated the diagnostic importance of combining CB with immunostaining using a mixture of p63 and cytokeratin 5/6 antibodies, and conventional smears of breast FNAB specimens.

The 169 lesions that were histologically confirmed to be malignant, including the 79 (46.7%) lesions that were detected using imaging, were analyzed. Based on our results, 12 of the 169 lesions reported as not malignant using conventional direct smear were diagnosed as malignant using CB. Histologically, these lesions were diagnosed as carcinomas with mild atypia or papillary structures. In addition, most of these lesions (i.e., 10/12, 83.3%) were detected by imaging alone. However, the number of unsatisfactory reports for histologically malignant lesions was significantly higher for CBs (38/169 specimens, 22.5%) than for direct smears (10/169 specimens, 5.9%), indicating that CB requires a larger amount of the aspirated specimens than that required for direct smears. Moreover, even after ensuring ample sample was allocated to CBs prior to preparing direct smears, we obtained an unsatisfactory rate for CBs from malignant lesions. Given that standard 20- or 22-gauge needles were used for FNAB, the size of the needle was not likely responsible for the insufficient amount of aspirated specimens. Meanwhile, 84 (49.7%) of the 169 lesions were detected by imaging or nipple discharge, thus, we postulate that the primary cause of the high unsatisfactory rates of CBs from malignant lesions was related to the high rates of non-palpable lesions. As such, the two preparations of conventional smears and CBs were complementary not competitive. Nevertheless, the inclusion of CBs with conventional smears resulted in efficient detection of malignant lesions in breast FNAB specimens, even for non-palpable lesions. In contrast, a prospective study from Nigeria has shown the potential of CBs prepared from the residual aspirate of 100 palpable breast tumors in a university teaching hospital [4]. Our study differs from theirs by involving a retrospective analysis of routine procedures (immunostaining in addition to HE staining) performed at a general hospital in a developed country, whereas their study was a prospective study, and immunostaining analysis was not performed. As such, some of the specimens include non-palpable small breast lesions that can only be detected by imaging. In addition, we routinely apply IHC staining of CB sections with a mixture of p63 and cytokeratin 5/6 antibodies.

IHC is a useful tool for providing additional information on FNAB specimens. In fact, several studies have highlighted the usefulness of IHC analysis to distinguish benign from malignant cytological samples [9]. More specifically, the combination of antibodies specific for p63 and a cytoplasmic marker, such as 34*β*E12 or cytokeratin 5/6, has proven useful for this purpose [10, 11], as we have also reported in tissues [5]. Additionally, IHC can be applied to CBs as well as direct smears and liquid-based cytology (LBC) specimens. However, compared with LBC or direct smears, IHC staining of CBs is typically more appropriate for routine clinical use as it does not require specific techniques or equipment. In addition, CBs can be stored in the same fashion as histology blocks and can be readily accessed to generate additional sections for further examinations.

Collectively, our findings indicate that combining CB with IHC and direct smears can further elucidate breast FNAB findings. Indeed, other studies have also shown that IHC staining of CBs improves diagnoses. For example, the application of CB with IHC for smears with thick-layered cell clusters or blood inclusion has proven useful for precise cytological diagnosis of breast tumors, particularly papillary lesions [12]. Furthermore, a case report involving two patients with lymphoma suggested that IHC staining of CB with the CD20 marker led to the diagnosis of diffuse large B-cell lymphoma for both cases [13]. Another breast myoblastoma case highlighted the potential of IHC and CB preparations in diagnosis [14].

Over the past 30 years, the use of FNAB for the diagnosis of breast diseases has decreased compared to that of CNB due to specific limitations of FNAB, including its lower sensitivity and specificity, and higher rate of unsatisfactory samples, as well as its inability to detect cancer invasiveness [1, 2]. According to a 1997 study, 50% of invasive non-palpable carcinomas were diagnosed based on direct smears via intensive examination to detect architectural features that were suggestive of invasiveness [15]. However, over the past 25 years, the size of non-palpable carcinomas has become considerably smaller due to an increase in the number of lesions detected by breast screening alone. Thus, new techniques are required to facilitate the extrapolation of pertinent information from breast FNAB specimens.

Since cytological specimens are considered to be unsuitable companion diagnostics for hormonal receptor and human epidermal growth factor receptor 2 (HER2) testing, and exhibit unsatisfactory rates in detection of non-palpable lesions [2], FNAB is being replaced by CNB, especially in developed countries. Moreover, an increase in the use of neoadjuvant chemotherapy and detection of lesions via breast screening has also contributed to this trend. However, CB resolves the issues associated with conventional preparation of cytology specimens, as they can be used for companion diagnostics [6, 16, 17]. Importantly, a recent Swedish study reports that 5–15 years after diagnosis of primary breast cancer, CNB-diagnosed patients exhibited significantly higher rates of distant metastases compared with FNAB-diagnosed patients [18]. We, therefore, postulate that the use of FNAB for diagnosis of breast diseases will become repopularized again in the near future.

In conclusion, this study introduced techniques used for the management of breast FNAB specimens at a general hospital in Japan. Combined use of CB with IHC and conventional smears resulted in the efficient detection of malignant lesions in breast FNAB specimens, especially of non-palpable lesions, that were detected upon imaging alone. Moreover, IHC staining of CB sections with a mixture of p63 and cytokeratin 5/6 antibodies provided more information than only HE staining. Thus, breast FNAB remains a useful procedure for evaluation of breast lesions, even in developed countries, when used in conjunction with CB preparation.

## Figures and Tables

**Table 1:** Cytological classification of lesion smears and corresponding histological findings.

Histological findings	Cytological classification of smears					Total
	Unsatisfactory	Benign	Atypia	Suspicious	Malignant	
Benign	5	1	7	1	0	14
Borderline	0	0	3	1	0	4
Malignant	10	5	25	36	93	169
Total	15	6	35	38	93	187

Atypia, atypia probably benign; suspicious, suspicious of malignancy.

**Table 2:** Classification of lesion cell blocks and corresponding histological findings.

Histological findings	Cell block classification				Total
	Unsatisfactory	Benign	Indeterminate	Malignant	
Benign	7	6	1	0	14
Borderline	3	1	0	0	4
Malignant	38	3	25	103	169
Total	48	10	26	103	187

**Table 3:** Classification of cytological smears and cell blocks of histologically confirmed malignant lesions.

Cell block classification	Cytological classification of smears					Total
	Unsatisfactory	Benign	Atypia	Suspicious	Malignant	
Unsatisfactory	6	2	8	10	12	38
Benign	0	2	1	0	0	3
Indeterminate	0	0	9	7	9	25
Malignant	4	1	7	19	72	103
Total	10	5	25	36	93	169

Atypia, atypia probably benign; suspicious, suspicious of malignancy.

**Table 4:** Clinical findings of lesions not reported as malignant using conventional smear but confirmed to be malignant using cell blocks.

Clinical findings	Cytological classification of smears			Total
	Unsatisfactory	Benign	Atypia	
Detected by imaging	4	1	5	10
Palpable mass	0	0	2	2
Total	4	1	7	12

Atypia, atypia probably benign.

## Data Availability

Regarding the collection of data, all data generated or analyzed during this study are included in this article. Further enquiries can be directed to the corresponding author.
